# Case of Excision of the Lateral Half of the Tongue

**Published:** 1871

**Authors:** George Buchanan

**Affiliations:** Surgeon and Lecturer on Clinical Surgery, Glasgow Royal Infirmary; Professor of Anatomy, Anderson’s University, etc., etc.


ARTICLE I.—*Case of Excission of the Lateral Half of the Tongue.* By GEORGE BUCHANAN. A. M., M.D.; Surgeon and Lecturer on Clinical Surgery, Glasgow Royal Infirmary; Professor of Anatomy, Anderson's University, etc., etc.

[Reported by WM. J. MORRIS, M.R.C.S., House Surgeon.]

CASE I.—J. T., aged 43, a Westmoreland farmer, was admitted to Ward XVII, on the 27th of April, 1870, and gave the following history:

About eleven weeks ago, he noticed a slight scratch on the right side of his tongue, which he supposed was due to some irritation from a carious tooth; this he describes as being at first "like a piece of wood splitting when drying." He complains of pain in the tongue, confined, till about three weeks ago, to that organ, but about that time it began to run up towards his ear in a darting manner. The pain in the tongue itself, he describes of a tingling character. At first the ulceration of the tongue made but slight progress; but about four weeks ago, it became greatly intensified. At present, the ulcer is confined solely to the right side, and the swelling does not extend to the middle line, nor to the submaxillary gland. It is greatly thickened, its edges undermined, and it is covered with a grayish white adherent slough. The submaxillary gland is not enlarged, nor are any of the glands in the neighborhood of the jaw. Patient has always enjoyed good health, and feels at present as well as ever he was.

*May 7th.*—Dr. Buchanan to-day performed the following operation: An incision was made through the centre of the lower lip, down to the hyoid bone, then the lower jaw was sawn through with a small straight saw; a piece of string was next passed round the divided sides, and given to two assistants to hold them apart; the muscular attachments of the affected side were then detached from the jaw. A ligature was passed through each side of the tongue, and thus the organ was well drawn out. An incision was then made down the centre of the tongue to the base, with a curved bistoury, and then, by a slight turn of the knife, the remaining attachment of the tongue was cut, and the affected side removed. There was not much hemorrhage, and this was promptly secured by ligaturing the lingual artery. The wound was then sponged with a solution of chloride of zinc (℥ii. to the ounce). A hole was bored through each side of the divided jaw, and strong silver wire was passed through, which was twisted three or four times, and so the divided sides were brought closely into apposition. The wound was then closed with fine silver sutures.

10*th.*—Patient is keeping very well, and is taking his beef-tea and brandy very well, being able to swallow without any difficulty. Pulse 102; no headache. The incision on the chin has almost completely healed.

11*th.*—Patient progressing favorably; pulse 100. Bowels are rather costive, and on that account has not been taking his food so well. To have 10 grains of calomel.

*12th.*—Patient is a great deal better; his bowels were freely opened, and he is taking his food better to-day. Pulse 98.

*13th.*—Dr. Buchanan to-day removed the sutures from the chin, as the incision has completely healed by first intention. There is a slight amount of suppuration at the lower part of the incision, but it is not of very much consequence. The jaw is beginning to get firm. Patient was up to-day for four hours; had been so for one hour yesterday. His diet has been principally beef-tea; but as he is getting tired of it, strong chicken-soup was substituted for it. The tongue is healing up very nicely.

*14th.*—To-day patient spoke a few words quite intelligibly; but as Dr. Buchanan does not wish him to speak, he keeps silent. The incision at the lower part of the chin has completely healed up. The silk around the teeth has come away, and has not been re-applied. He enjoys his chicken-soup very much, and he is able to swallow without any inconvenience.

*17th.*—Patient is improving rapidly. The surface of the tongue has almost completely healed, and the jaw is becoming firmer. His general health is excellent, and he takes his food very well. He can speak quite well, perfectly intelligibly to his wife, but to others not quite so, owing to his dialect.

*28th.*—Since last note, patient has greatly improved; there is no discharge from the mouth; salivation has completely stopped. The jaw is getting quite firm, and the silver wire through the symphysis was removed. His speech is quite intelligible. Dismissed, cured.

Case II.—Miss S., æt 48, single, admitted to Ward XXV, of Glasgow Royal Infirmary, on 16th May, 1870. Patient has an ulcer on the right side of her tongue, of which she gives the following history :

In August last, she observed a small pimple at the right side of the tongue, near the tip, which gave her no uneasiness at the time; but as it was gradually getting bigger, she consulted her medical man, who advised her to have a carious tooth removed. This was done, but it did not, in any way, influence the affection; it gradually and slowly got larger, till at the present time it is as large as a small nut. About three weeks ago, she applied nitrate of silver to it, and this was followed by ulceration, so that there is now a dirty excavated sloughy ulcer on the right side of the tongue about half an inch in diameter. There has been a great deal of pain in the organ during the whole of the winter, which was greatly aggravated by any exposure to cold; but latterly, this pain has been getting more severe, and she states that for two or three days befor admission it has been most excruciating. The pain, she describes as of a stinging character, shooting up to her ear. There is no enlargement of the submaxillary gland, or of any of the glands in the neighborhood of jaw.

There is no history of cancer in the family, but she says that her father's family, fourteen in number, almost all died of decline.

*May 18th.*—To-day, patient was put fully under chloroform, and Dr. Buchanan performed the following operation: Two strong ligatures were passed, one through the healthy, and the other through the diseased side, and so the tongue was well drawn out. An incision was then made down the centre of the organ almost to the base. The chain of an ecraseur was then passed beyond the disease and it was removed, There was a little bleeding from the lingual artery, but it was promptly secured by ligature.

11 P. M.—Patient is very comfortable, not having much pain. No hæmorrhage.

*19th.*—Patient is very comfortable; no hæmorrhage. She is taking her beef-tea and brandy very nicely, in small quantities, often repeated.

*20th.*—Patient has a slight headache this morning; as her bowels are costive, she is to have ℥ss. of castor oil.

*21st.*—Headache is gone; bowels well opened. She feels quite well; appetite excellent; pulse good.

*23d.*—Patient is steadily improving. As there is a slight discharge, which is accompanied with fœtor of the breath, she is to wash her mouth often with Condy's fluid. Patient was up today.

*25th.*—There is a little pain on the tip of the tongue; but as it is cicatrizing, it is probably due to that.

*27th.*—Patient is quite well, and was out to-day. The wound of tongue has almost completely healed up.

*June 2d.*—Patient was dismissed to-day; wound entirely healed; she speaks intelligibly.

*Re-admitted June 13th*, 1870.—It was stated in the report of the 25th May, that patient felt a slight pain at the tip of the tongue, which was then thought to be solely due to the cicatrization; but upon her return now, that supposition is found to be incorrect, as the discease has shown itself at the site of the cicatrix. It is perfectly hard and slightly indurated, with a slight fœtid discharge. The glands of the neck are still free from any enlargement.

*18th.* — To-day, Dr. Buchanan removed, by the operation detailed in Case I., the whole of the right side of the tongue, encroaching in his median incision slightly to the left side, so that there was more hæmorrhage from this case than usual, as both lingual arteries were cut, and also because the submaxillary of the left side was abnormally large.

11 P. M.—Patient has had a very good day; being asleep for the greater part of the time. There has been very little oozing from the wound. Pulse 92.

*19th.*—Patient slept very well last night, and does not feel so much pain in the tongue to-day. She has been able to swallow a little milk and beef-tea during the night. She complains of a headache; pulse good.

*20th.*—Patient has had her bowels freely opened, and feels herself considerably relieved, the headache having gone altogether. She is able to swallow better to-day, but has still a little difficulty in doing so. She feels the ice very grateful.

*21st.*—Patient is still keeping very well; the oozing has stopped entirely; but there is a good deal of salivation.

*23d.*—The sutures from the chin were removed to-day, union being perfect by the first intention, except at the lower part of the incision, where there is a slight discharge, so that the sutures are to remain there for a day or two longer. The odour of the discharge being rather fœtid, patient is to have her mouth syringed often during the day with a solution of the chlorate of potash (ʒii. to ℥vii.) ; but as she says this smarts her, a week solution of Condy's fluid was substituted.

*25th.*—All the sutures were removed to-day, union being perfect, except at the lower angle, which still discharges a little.

*28th.*—Patient was up to-day for a short time, and felt herself better for it. The wound in the tongue itself is looking pretty well, but it is covered with a grayish white fur, which, despite the washing of the mouth frequently with the Condy's solution, still continues.

*July 6th.*—Patient has steadily improved since last note, and is now able to speak rather intelligibly. Salivation, which throughout has been rather profuse, is now a great deal less. The jaw is becoming firmer daily.

*19th.*—To-day, Dr. Buchanan removed the wire from the jaw, as the divided sides had become quite firm. There is still a fur on the tongue, which has now adapted itself to the centre of the mouth.

*August 5th.*—Nothing of note has occurred since last report, and to-day patient was dismissed.

Caee III.—Colin M'Lachlan, æt. 49, fireman, married, admitted to Ward XVII. of Glasgow Royal Infirmary, 27th May. Patient says that about two months ago, he noticed a small pimple on the right side of the tongue, about the size of a pin's head, which he scratched off with his finger-nail, after which it began to get bigger, and then an ulcer made its appearance, which was hard and indurated, and which has gradually got bigger till the present time. At first it was not very painful, but, after the ulceration commenced, he says it was very much so, especially when it came in contact with his teeth. He describes the pain as being only for a short time (not continual), and of a shooting "stooning" character, up to the ears, principally the right, but occasionally also to the left. His teeth are not in any way carious, and are perfectly sound, and he says he was never troubled with toothache. He has been a rather heavy smoker, as much as two or three ounces of tobacco a week ; his pipe being a "short, black cutty."

*Family History.*—None of cancer or of phthisis. His father died at the age of 96 years.

On examining the tongue, there is found to be an ulcer invading principally the sublingual gland, but also extending to the tongue on the right side. It is rough to the touch, and decidedly indurated. The extent of surface involved is altogether about the size of a five-shilling piece; and the patient says that there is a good deal of discharge from its surface, which is rather fœtid. The glands under the jaw and the neck are perfectly free from any enlargement. Patient's health is as good as ever it was, and he feels himself quite strong. Owing to the offensive smell of the discharge, to have a wash of chlorate of potash, ℨii. ad ℥viii.

*June 11th.*—Dr. Buchanan performed the operation before detailed, and there was nothing unusual about the operation. The hæmorrhage was very slight, owing to the promptness with which the vessels were secured by ligature. The whole cut surface in this as well as the other operations, was well sponged with a solution of chloride of zinc, (ℨi. to ℥i. of water).

11 P. M.—Patient has not had much pain since the operation, and slept for about three hours in the afternoon. To have 40 drops of laudanum by injection to-night. Pulse 86; very good.

*12th.*—He slept a few hours during the night, and does not complain of much pain in the tongue. There has not been much oozing, and what there is, is only slightly sanguineous. He is to suck ice constantly. He was able to swallow some beef-tea, with a little brandy, very well. Pulse 94.

*13th,* 10 A. M.—Patient slept almost the whole of last night. Does not complain of much pain in the tongue; oozing considerably less; continue ice, etc. Dr. Buchanan saw him in the afternoon; and as there was a little fœtor of the breath, he ordered the mouth to be syringed frequently with a solution of chlorate of potash.

*14th.*—Patient is improving very nicely; is taking his beef-tea, with some brandy, very well, and does not complain of much difficulty in swallowing, nor of pain in the tongue.

*15th.*—Patient is progressing very favorably. There is not much discharge from the surface of the tongue, as it is often washed with the chlorate of potash solution.

*16th.*—Dr. Buchanan removed the sutures from the chin, the wound having healed by first intention, except at the lower angle, where there is a slight discharge; and so a linseed-meal poultice is to be applied to it. Patient is keeping very well, and does not complain of any pain in the tongue.

*18th.*—Patient got up to-day, and is able to speak very intelligibly. He feels quite well; is able to swallow very well; salivation is becoming less.

*24th.*—Since last note, patient has progressed very favorably, being able to speak quite intelligibly. He takes his food well, and has no difficulty in swallowing. The jaw is becoming firm.

*July 8th.*—The wire from the jaw was removed; union between the two sides being perfectly firm.

*9th.*—Dismissed well.

*P. S.*—It may be interesting to the readers of this Journal to know, that a patient on whom I operated in May, 1865, is still well, and free from any return of the disease. The case is related in the number of the *Edinburgh Medical Journal* for November, 1865.—*Ed. Medical Journal.*

